# The effects of six months of the adapted Otago Exercise Program on cognition and falls relative to usual care alone among people living with dementia in residential care facilities: A pilot feasibility randomized controlled trial

**DOI:** 10.1002/alz.087957

**Published:** 2025-01-09

**Authors:** Deborah A Jehu

**Affiliations:** ^1^ Augusta University, Augusta, GA USA

## Abstract

**Background:**

There are no evidence‐based exercise guidelines to reduce falls in people living with dementia (PWD). The purpose of this pilot randomized controlled trial (RCT) was to determine the 1) feasibility and acceptability, and 2) effect of exercise on cognition and falls (exploratory) relative to usual care alone among PWD.

**Methods:**

We randomized PWD to the exercise (*n* = 21) or usual care group (n = 21) at two residential care facilities in our pilot parallel, assessor‐blinded RCT (1:1) [NCT05488951]. We examined feasibility (recruitment, data collection, dropout rates, adverse events, adherence) and acceptability (satisfaction). Our adapted physical therapist‐led Otago Exercise Program consisted of 1 hour of strength, balance, and walking 3x/week for 6 months in groups of 5‐7 PWD. At baseline and 6 months, participants completed the Color‐Word Stroop 3‐2 (primary), Montreal Cognitive Assessment (MOCA); Trail‐Making test, Digit Span Forward/Backward; Benton Judgment of Line Orientation; Boston Naming Test; Rey Auditory Verbal Learning Test; Digit Symbol Substitution Test, and the Health Utilities Index‐3 (HUI‐3). We pulled falls monthly from medical charts. We performed intent‐to‐treat (ITT; n = 42) and per protocol analyses (n = 9 with ≥2x/week exercise adherence, n = 21 usual care), controlling for age, race, sex, and the MOCA.

**Results:**

Recruitment and enrolment were completed in 7 months (36% female; 17% African Americans; Age:82.1±8.1years; MOCA:10.2±5.9points). The attrition rate was 19.0%, exercise adherence was moderate (60.2±34.5%; 47/78 exercise sessions), and satisfaction was good (4.2/5 points). The trial established protocol safety, with no adverse events related to the intervention. The ITT analysis revealed no differences in the Color‐Word Stroop (primary), but better executive function (Digit Span Backwards) following exercise relative to usual care (p<0.05). The per‐protocol analysis revealed better executive function (Digit Span Backwards) and HUI‐3 following exercise relative to usual care (p<0.05). There were no effects on falls (exercise:13 falls, n = 8 recurrent, n = 1 injurious; usual care:13 falls, n = 10 recurrent, n = 4 injurious).

**Conclusion:**

This pilot RCT was feasible and acceptable. Any amount of exercise appears to improve executive function, with at least 2x/week exercise also benefiting quality of life among PWD. These findings may have implications for targeted treatment strategies and inform a larger trial powered for falls in PWD.

